# Income inequality in the uptake of environmentally friendly products

**DOI:** 10.1016/j.isci.2025.112277

**Published:** 2025-03-25

**Authors:** Martina Maglicic, Vítor V. Vasconcelos

**Affiliations:** 1 CeNDEF, Amsterdam School of Economics, University of Amsterdam, Roeterstraat 11, Amsterdam 1018 WB, the Netherlands; 2 Institute of Environmental Science and Technology, Autonomous University of Barcelona, Campus UAB Bellaterra 08193 Cerdanyola del Vallès, Spain; 3 Computational Science Lab, Informatics Institute, University of Amsterdam, Lab 42, Amsterdam 1090 GH, the Netherlands; 4 POLDER, Institute for Advanced Study, University of Amsterdam, Oude Turfmarkt 147, Amsterdam 1012 GC, the Netherlands

**Keywords:** Environmental policy, Social sciences

## Abstract

The uptake of environmentally friendly products is unequal and income-dependent. Whether solar panels or electric vehicles, lower income groups are often locked out of the benefits they offer. Worse, policies encouraging the adoption of environmentally friendly products have replicated or even exacerbated inequalities. We experiment with analytical and agent-based models to examine mechanisms driving this inequality trap and explore policy solutions. Considering economic factors and social desirability aspects of green products, we compare adoption across income quartiles. Our findings indicate higher-income groups are much more sensitive to social desirability, including environmental awareness and peer and status effects, while lower-income groups are primarily concerned with financial constraints. Additionally, social tipping points can either enhance or hinder adoption, depending on the level of economic inequality. Without a targeted approach addressing the financial barriers of lower-income groups alongside community-wide interventions achieving a just energy transition will remain a challenge.

## Highlights

• Income inequality can both increase and decrease adoption
• Lower income groups are restricted by financial factors
• Higher income groups are motivated by peer effects, status, and environmental concern
• Targeted financial support for lower-income groups is needed for equal adoption

## Graphical abstract

**Figure:**
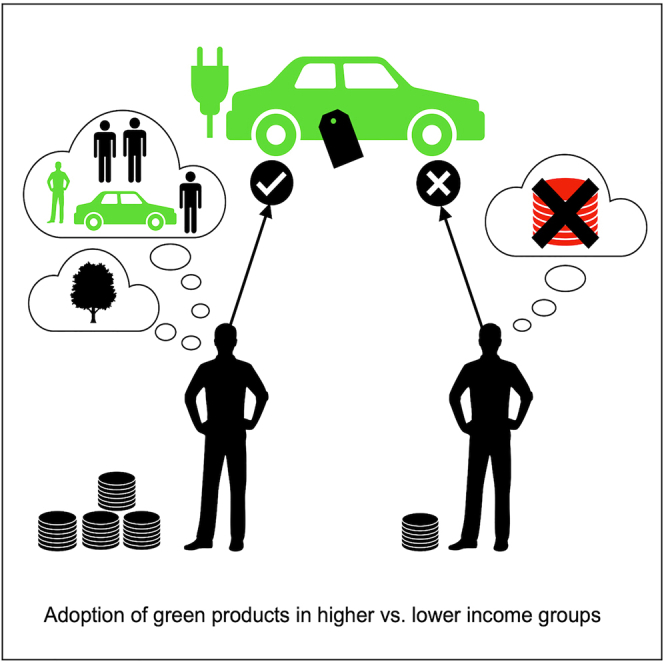

## Introduction

The transition away from fossil fuels as the main energy source to renewable technologies such as solar, wind, or hydropower intersects various fields—environmental science, psychology, economics, and public policy—each playing a role in understanding and influencing the global shift toward sustainability. Economic factors, social norms, and policy environments collectively affect consumer and producer behaviors.^1^^,^^2^^,^^3^^,^^4^^,^^5^^,^^6^ For instance, the decision by consumers to adopt solar panels, heat pumps, or electric vehicles is pivotal in this transition.^7^^,^^8^^,^^9^^,^^10^ Historically, these choices are influenced by various policies designed by governments aiming to meet the 2030 climate objectives. These policies focused on financing instruments, including feed-in tariffs, subsidies, marketing campaigns, taxes, infrastructural policies, information campaigns, and rebates.^11^^,^^12^^,^^13^^,^^14^ Despite these efforts, a multitude of barriers such as awareness, product price, social influence, switching costs and motivation persist, decelerating the pace of the energy transition.^15^^,^^16^^,^^17^

**Figure 1:**
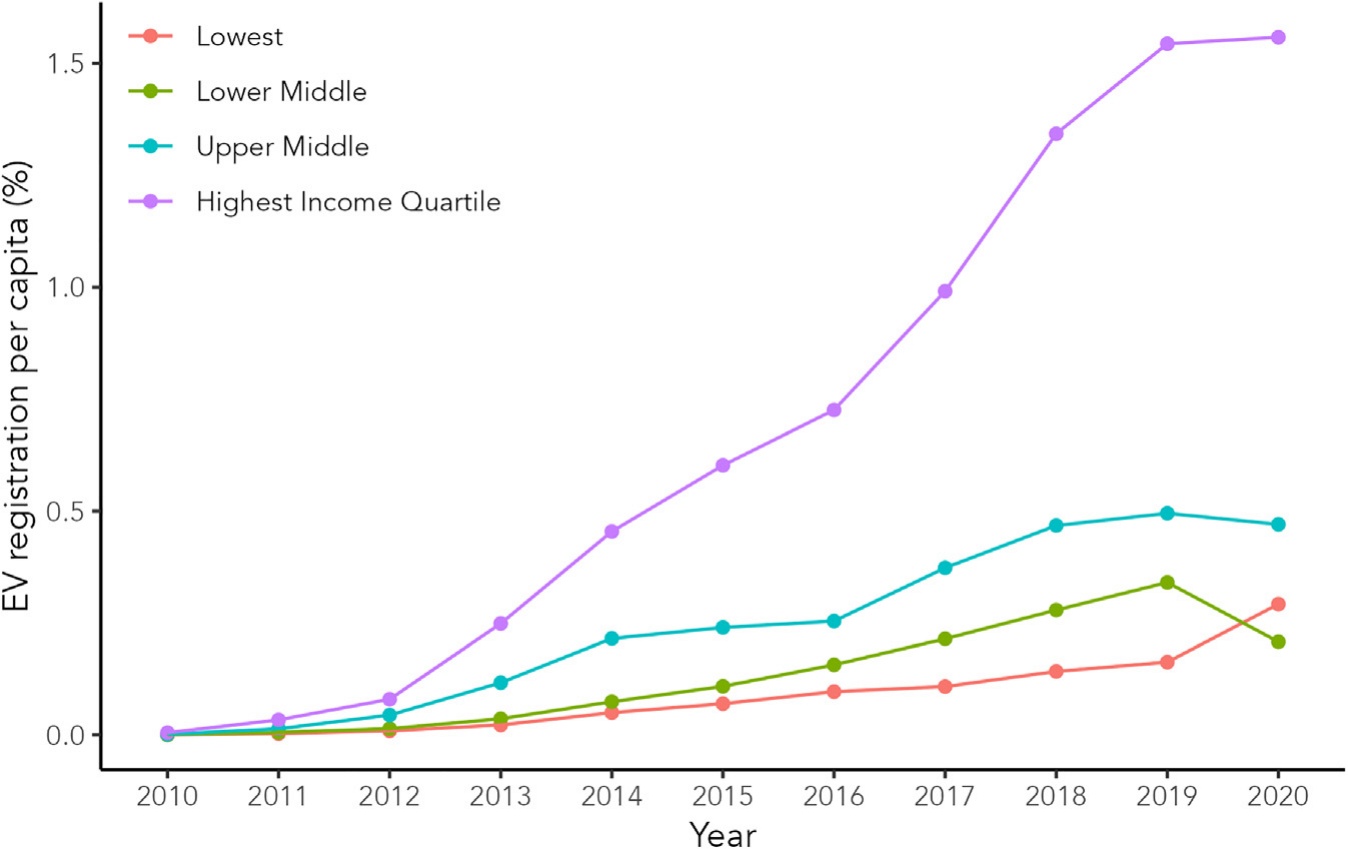
Higher income regions adopt EVs earlier and to a greater degree The figure shows electric vehicle (EV) registration per capita by county in California. We divided counties into four income groups based on the GDP (Gross Domestic Product), and computed the average number of EV registrations per capita for each quartile. We observe a cascading effect, where EVs first gained traction in higher-income neighborhoods, and adoption then trickled down the other income groups. The trend appears stable over time. The data comes from the US Bureau of Economic Analysis^26^ and the Atlas EV Hub.^27^

The impact of barriers—whether they be cognitive, social, product-related, or economic—on behavior is not homogeneous across the population. The uptake of environmentally friendly products among consumers from different income groups has been unequal, with higher income groups adopting environmentally friendly products to a larger degree and reaping more of their benefits.^3^^,^^18^^,^^19^ Figure 1 shows the registration of electric vehicles in California: higher-income counties have adopted electric vehicles earlier and to a greater degree than their lower-income neighbors. This has not only been found for electric vehicles,^20^^,^^21^ where 90% of all tax credits for the purchase of an EV went to the top income quintile, but also for solar panels,^22^^,^^23^ where higher-income households are exponentially more likely to own them. Similarly, efficient appliances^24^ exhibit income-driven disparities, as lower-income households are significantly less likely to choose the green option, often due to high implicit discount rates that make future energy savings less appealing compared to the higher upfront costs. Even energy-efficient light bulbs^25^ show unequal adoption, driven by their unavailability and higher upfront costs in stores located in lower-income areas. These examples highlight the pervasive role of income inequality in limiting access to and adoption of energy-efficient technologies across multiple domains. While it is clear from the figure that the state of California has implemented a successful combination of policies, a key question remains, which is what policies and factors drive such cascading adoption-behavior.

Typically, lower-income groups face different circumstances and opportunities than higher-income groups. They are more financially constrained, have limited access to infrastructure and information, and suffer from higher stress levels that can impair decision-making processes.^28^^,^^29^ Furthermore, monetary scarcity increases risk aversion and present bias, neither of which favor switching to a green alternative.^30^^,^^31^ On an individual level, low income can, therefore, suppress product diffusion and lead to non-optimal responses to climate stressors, even though these individuals are more vulnerable to adverse consequences of climate change in the first place.^18^^,^^32^^,^^33^^,^^34^ On a societal level, the effects of income inequality on adoption are still poorly understood, especially as they interact with social aspects of adoption. While there is substantial research on the role of inequality in adoption and its interaction with social aspects (as reviewed previously) in the context of the individual, to the best of our knowledge, the literature has not focused on the emergent aspects of green technology adoption in the context of income inequality. Specifically, how individual-level behavior, influenced by the behavior of others, aggregates into collective adoption patterns across income groups remains poorly understood. This emergent perspective is critical for understanding and addressing inequalities in the energy transition.

**Figure 2:**
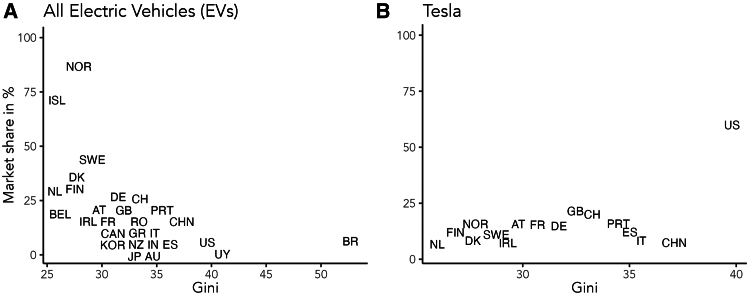
Income inequality can both increase and decrease adoption of EVs The two panels show the electric vehicle (EV) market share by country as a function of income inequality (Gini), (A) for Tesla cars only, (B) for all EVs. We observe a difference between the market share of all EVs and Tesla cars specifically. While the market share of all EVs seems to decrease with rising inequality, we observe an increase in market share up to a threshold of a Gini coefficient of 32.6, followed by a decrease, for Teslas. The data comes from the World Bank,^37^ Canary Media^38^ and the World Population Review.^39^ The figures are from 2022.

Figure 2 shows that income inequality affects product adoption behavior in diverse ways, and it has a heterogeneous and non-monotonic impact on adoption depending on the product. While Figure 2 seems to suggest that increasing inequality is associated with a decreasing adoption ratio for electric vehicles, for Tesla, specifically, a premium electric vehicle, the effect is not as clear-cut. In fact, especially ignoring the US data, increasing income inequality seems to entail higher adoption up to a certain point. The literature on the consumption of status goods, which finds that the higher the inequality, the more popular luxury goods become, may explain half of this result.^35^^,^^36^

Understanding the impact of income inequality on the adoption behavior of different income groups is crucial, especially when these interact through social and political mechanisms. Many policies aimed at increasing the uptake of green products have been criticized for replicating or even exacerbating existing economic inequalities. This includes both financial incentives and policies striving to increase the social desirability of green products. For example, the feed-in tariff in the UK is financed by lower-income groups through proportionally higher energy bills while mainly benefiting higher-income groups.^40^^,^^41^^,^^42^ Similarly, subsidies have been shown to lead to unequal adoption when a high up-front cost is only later reimbursed.^43^ This is also the case for interventions relying on peer effects, which can reinforce barriers from financial constraints by concentrating information and awareness of beneficial policies in wealthier regions.^41^ In fact, some have even argued that climate policy and inequality goals are directly opposed to each other.^44^ As energy systems have societal values embedded in them, a key concern from the energy justice movement has been that the energy transition aggravates existing social issues, such as poverty, corruption, or crime, and undoes or hinders decades of progress.^45^^,^^46^ It has been argued that the energy transition should ideally help resolve social issues rather than hinder them. As poorer households consume less than their wealthier counterparts, they also emit less. They contribute less to climate change and miss out on the benefits of environmentally friendly products if they do not adopt them.^3^^,^^47^^,^^48^^,^^49^ This can be problematic because the less wealthy frequently benefit the most from these innovations, as they can help save money and time, something that poorer households often lack.^31^

To achieve a “just” transition, with equitable access to clean technologies and their benefits,^50^ we need to understand how different mechanisms and policies drive (un)equal uptake and how governments can not only boost the adoption of environmentally friendly products but also do so fairly without leaving behind lower-income groups. It is essential to understand how inequality replicates despite (or even because of) policy interventions and what is needed to allow lower-income groups to also participate in the transition. Contrary to most empirical studies, which primarily examine individual-level behavior in isolation and often focus on the effects of various policies on adoption decisions without considering how these behaviors aggregate in a social context or income groups, we choose to focus on the aggregate effects of different mechanisms on societal outcomes scaling up the direct effects on the individual to generate new hypotheses. Our study therefore differs by exploring the emergent dynamics of adoption, specifically how interactions between individuals and income groups influence collective patterns, with a particular emphasis on the role of income inequality. This approach highlights not only individual adoption barriers but also systemic factors that shape unequal outcomes in the energy transition. Therefore, our paper aims to answer the following research questions.

RQ1: How does income inequality affect adoption behavior across income groups?

H1: Income inequality changes adoption rates across income groups differently.

H2: The larger the inequality, the bigger the gap in how income groups respond to changes in social desirability and financial factors.

RQ2: Which individual-level factors drive adoption behavior across income groups, and how can these be exploited to boost adoption levels, particularly for lower-income groups?

H3: Social desirability factors, such as product visibility and environmental concern, are more effective in boosting adoption behavior among higher-income groups.

H4: Financial constraints play a larger role in limiting adoption behavior among lower-income groups.

We develop an analytical model to identify different scenarios and integrate two categories of factors that have been shown to affect uptake: economic factors, which includes product price and consumer income, and social desirability of the green product, including environmental concern levels in the community and visibility of the product, which enables peer influence. Taken together, the economic factors determine the available budget for the purchase of environmentally friendly products. Environmental concern increases the desirability of switching to a green(er) alternative. Having a green product that is visible allows one’s peers to easily spot the purchased product and imitate the purchase. For example, a Tesla would be a highly visible environmentally friendly product, while a heat pump is not.

We extend the model into a computational model that addresses temporal dynamics. We study the adoption levels of environmentally friendly products by income quartile. Quartiles were chosen for the analysis to balance simplicity and computational feasibility while capturing meaningful income-based disparities in adoption behavior. Quartiles provide a manageable yet representative segmentation of income groups, allowing us to explore heterogeneity without overwhelming the computational model with excessive granularity. This approach ensures the model remains interpretable while adequately reflecting income-driven adoption dynamics.

Building on classic diffusion approaches while integrating an agent-based framework enables us to capture both the well-established mechanisms of product uptake and the rich heterogeneity observed in real-world adoption patterns. Classical models (e.g., a study by Bass^51^) offer a foundational understanding of how innovations spread through populations, yet they often assume uniform decision-making and overlook critical factors such as income disparity and nuanced social influences. Recent advances in agent-based modeling (ABM) have made it possible to relax these assumptions and more accurately represent decentralized decision-making processes, social networks, and heterogeneity in agent attributes.^52^ In this context, ABMs have gained traction in economics and finance for studying complex market dynamics, policy interventions, and systemic risk in ways that traditional models cannot fully capture.^53^ Our study contributes to this evolving literature by explicitly modeling the interplay between income inequality, environmental awareness, and status-seeking behavior, demonstrating how classical diffusion mechanisms can be extended through ABM and global sensitivity analysis^54^^,^^55^ to yield insights into equitable and inclusive pathways for green technology adoption.

We find that the most important factors differ by quartile. While the top two income quartiles respond best to increasing social desirability of the product, which motivates their purchase and are less sensitive to economic aspects, the adoption of lower income groups is almost exclusively governed by the latter, specifically purchase price and inequality. Unless easily accessible financial support is provided, especially once the adoption cascade has started, it is difficult to meaningfully boost their uptake of environmentally friendly products or ensure equal uptake. The findings have strong implications for policymakers who should be wary about “fit-for-all” policies that seemingly do very well in increasing product diffusion, as they are likely to have boosted adoption for the wealthy and left behind the rest. Instead, targeted and sequential support for lower-income groups is needed to complement community-wide policies.

## Results

We consider a population of utility-driven decision-making individuals who weigh several factors, namely price, environmental concern, product visibility, and default effects as well as different shape parameters for the utility. Table S1 in the supplemental information shows the set of validation statements underlying these factors and mechanisms. Agents are heterogeneous in their income. In the analytical model, individuals are purely rational and compare themselves with those in higher income brackets—their reference group—and we solve for equilibrium. We consider an extended computational model that allows for a range of reference groups and errors and considers temporal dynamics.

### Inequality is paramount for adoption, but it can be both a hindrance and a booster

**Figure 3:**
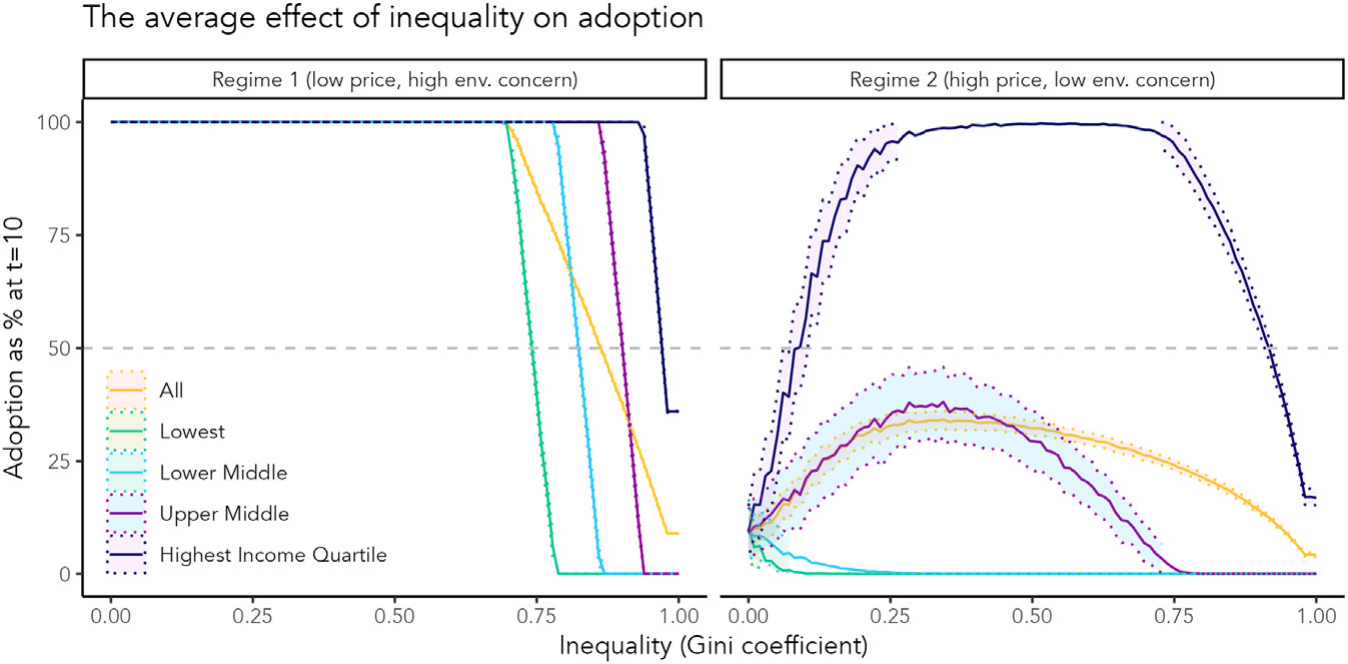
Income inequality effect on the adoption ratio Two regimes of the effect of inequality that differ in product price (R1: p=0.01, R2: p=1) environmental concern (R1: $\bar{d}=0.8$, R2: $\bar{d}=0.1$), and *θ* (R1: θ=3.32, R2: θ=2.73). Income inequality was systematically varied across both regimes. The rest of the parameters were the same in both scenarios (β=5, v=1, n=100, ϵ=6, γ=−1, σ=6, default effect =0), and each parameter combination was run 200 times. The dotted bands indicate standard deviations from the average adoption ratio, represented by solid lines.

In Figure 3, we distinguish between two regimes based on findings from our analytical model (for mathematical proofs, please see the supplemental information). We look at the adoption ratio across the population and for different income quartiles at an arbitrary time point. Regime 1 is characterized by a low purchase price and high environmental concern, while a higher purchase price and lower environmental concern characterize Regime 2 (for the conditions, please see the supplemental information).

Overall, income inequality affects adoption behavior non-linearly and differently across income groups. Furthermore, income inequality can either increase or decrease the overall adoption ratio, but nearly always increases or maintains adoption inequality and only reduces inequality in adoption by reducing total adoption. Only a regime of low cost and low inequality leads to uniform high adoption levels. The conditions that lead to these differences in outcomes are 2-fold: one determinant is product price and the other is environmental concern. Low price combined with high concern leads to negative effects of inequality on adoption. On the other hand, in a high price and low concern scenario, top adoption rates peak around low to medium inequality and decline after.

In Regime 1, increasing inequality leads to a transition in collective adoption behavior from 100% to 0% adoption. In the lower-income quartiles, this transition happens for lower inequality levels, increasing adoption inequality. This is driven by financial constraints, which make the lowest earners extremely unable to purchase the environmentally friendly product even when their wealthier reference group does. On the other hand, higher income groups keep adoption at 100% for higher levels of inequality. Figure 3 shows this transition for one set of parameter values. In this case, the adoption ratio starts diverging from 100% and decreasing to 0% at medium to high inequality levels (0.707 globally), depending on the income group. The average rate of change during the transition ranges from −13% for the highest income group to −35% for the lower middle-income group. Note that the parameter space at which the transition in adoption behavior takes place differs across scenarios and so does the rate of change.

Regime 2 is characterized by a higher purchase price and lower environmental concern. Contrary to Regime 1, the widening wealth gap spurs adoption by wealthier individuals; it increases their purchasing power and allows them to buy environmentally friendly products and trigger a (potentially contained) cascade. This effect is reversed when inequality reaches a point where only a few individuals possess most of the wealth. Then, increasing income inequality still triggers the cascade of adoption from higher to lower-income groups, but if pushed too far, it can also hinder adoption of the low- and medium-income groups due to their growing financial constraints. In this regime, higher income inequality can increase overall adoption but at the cost of widening adoption inequality.

Furthermore, we observe an inverse relationship between outcome adoption inequality and adoption ratio in Regime 1, but, in Regime 2, a higher adoption ratio is accompanied by higher adoption inequality.

### Successful strategies to boost adoption levels are heterogeneous across income groups

Due to the importance of income inequality discussed in the previous section, income groups are heterogeneous in responding to changes in different factors. Based on our computational and analytical models, we have identified three distinct types of behavioral responses among individuals and the exact conditions of their classification, determined by their income levels and the cost of environmentally friendly products. Type A individuals are non-adopters, who never adopt due to the entry cost. Type B individuals are universal adopters, who always adopt. Finally, type C individuals are trend followers, who adopt if others do. For a mathematical definition of the types, see Theorem S5 in the supplemental information. Different income quartiles consist of varying proportions of these behavioral types, which allow us to interpret the contribution of various factors to the adoption ratio across quartiles.

**Figure 4:**
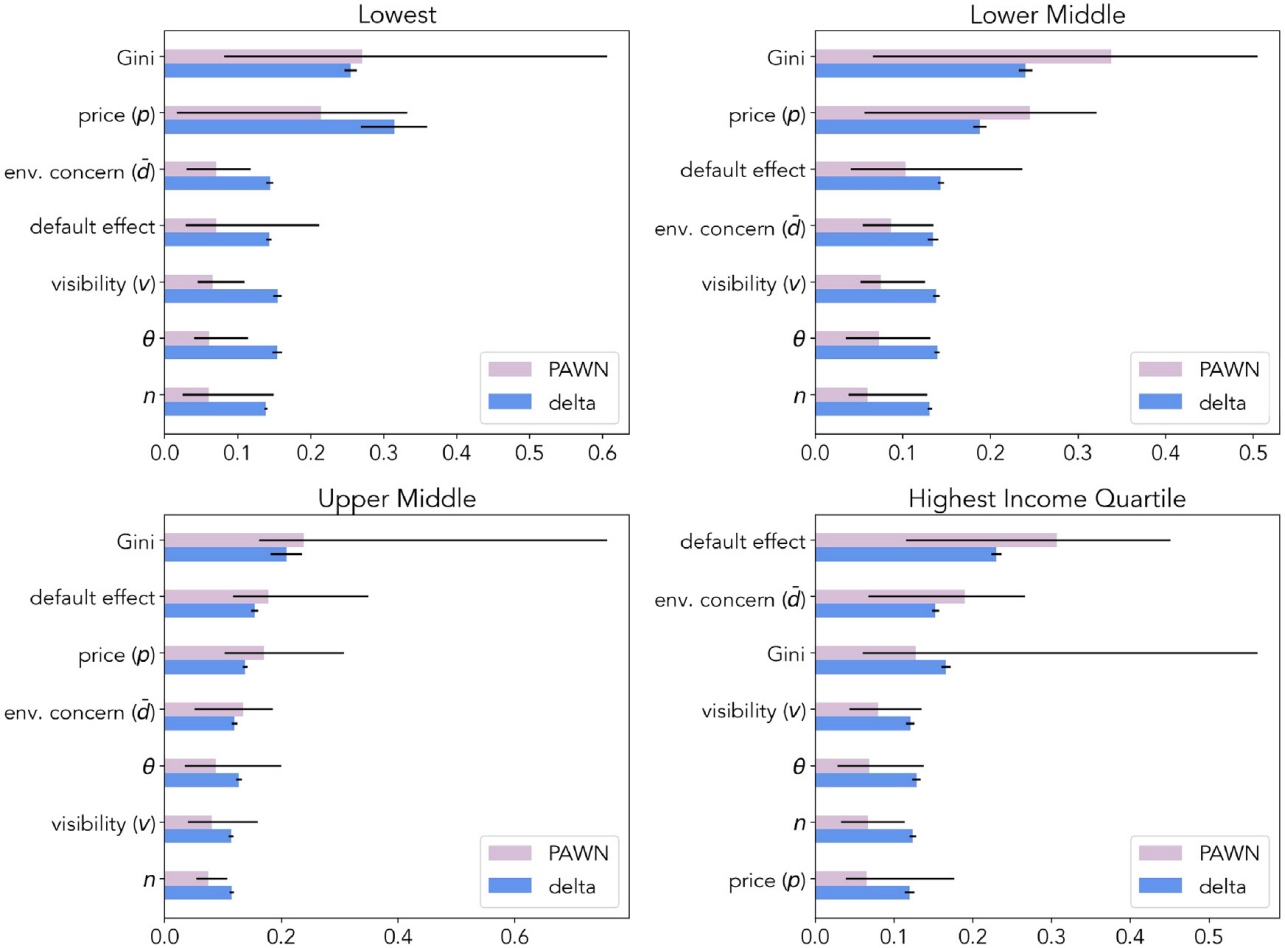
Income inequality drives adoption inequality, and social desirability primarily boosts adoption for higher income groups The figure shows the main effects of model parameters by income quartile. Results from 2 analysis approaches are compared: PAWN and Delta. Bars are ranked based on results from PAWN. Bars indicate the estimated effect size and, in the case of PAWN, the median effect size. Black lines show confidence intervals for Delta. For PAWN, these are minimum and maximum estimated effect sizes. Gini coefficient refers to the level of income inequality, visibility to the conspicuousness of the product, *θ* regulates sensitivity to product price, and *n* to the number of agents.

Figure 4 shows the importance and effect sizes of different parameters by income quartile. We use two key global sensitivity analysis methods (i.e., when varying all parameters simultaneously), PAWN and Delta, to discern the impact of changing various factors on the adoption of environmentally friendly products in each quartile. The PAWN method is designed to capture the change in the cumulative distribution function of the product update induced by each parameter. Meanwhile, the Delta quantifies the change in model output variance attributed to variations in each parameter. These techniques allow us to effectively illustrate the effects of changing Gini coefficients, price, environmental concerns, default effects, and product visibility for the range of all possible values of the other parameters. The results reveal notable differences in the effect sizes and characteristics of these parameters across income quartiles. The outcomes in the highest quartile are the most sensitive to the default effect, representing the cost-unrelated inertia of changing strategy, followed by environmental concern. Adoption across quartiles is the most sensitive to inequality (Gini), being the parameter with potential maximum effect also for the highest quartile. Price ranks highest for the lower quartiles. Overall, social desirability factors demonstrate a significantly stronger influence in the highest income quartile compared to the lowest, where affordability constraints diminish their importance. Meanwhile, environmental concern, product visibility, and default effects increase with importance at higher income levels. The following subsections delve into these results in further detail.

#### Effects of factor changes on wealthiest quartile

Individuals in the highest income quartile consist mainly of universal adopters (type B), who always adopt, and trend followers (type C), who adopt if others do. Thus, the adoption of this quartile will depend on the fractions of each of these types and the effective behavior of trend followers in that quartile. For both types, cost is not a significant barrier and income inequality can, in fact, benefit them when the purchase price is high (see Section 2.1). The average level of environmental concern determines the fraction of each of the two types, with higher concern increasing the fraction of universal adopters. Trend followers require product visibility for adoption.

When varying these factors, the most important factor determining the adoption ratio for the highest income quartile is the strength of the default effect. This can be thought of as comprising non-monetary switching costs, such as the effort and time needed to make the switch. These disproportionately affect higher-income groups, where affordability is not a barrier. For these groups, the default effect becomes a critical determinant of adoption as they weigh the convenience or inertia associated with the default against the minimal perceived cost of switching. Conversely, for lower-income groups, financial constraints overshadow non-monetary switching costs, making the default effect less significant as affordability remains the primary barrier to adoption. The lower the income group, the less important the default effect becomes, as even with low switching costs, the consumers are unable to afford the product. Convenience and ease of switching becomes secondary. Thus, non-monetary switching costs can be understood as determinants of differences in the default effect’s importance across income groups, as these costs are more salient when financial constraints are less pronounced.

Environmental concern is the second most important factor determining the adoption ratio for the highest income group but is only in third place for all income groups combined (see Figure 4). Similar to visibility, its importance increases with income. This is the case because the fraction of trend followers increases and financial constraints play less of a role. Its positive effect manifests itself in enlarging the regime of full adoption, where it counteracts the negative effects of financial parameters such as purchase price and inequality and speeds up adoption. Again, since the fraction of trend followers increases with environmental awareness, high-income groups are the ones most likely to be affected by changes in environmental awareness.

Product visibility affects adoption behavior when adoption is partial. In the parameter space of partial adoption, high-income trend followers can still purchase the products, but their behavior is susceptible to changes in social desirability factors (visibility and environmental concern). Therefore, increased product visibility predominantly benefits top earners and, thus, also contributes to widening inequalities. Figure 4 shows that while tends to rank low in importance for middle and lower income groups, it likely has a stronger impact on the highest income quartile.

**Figure 5:**
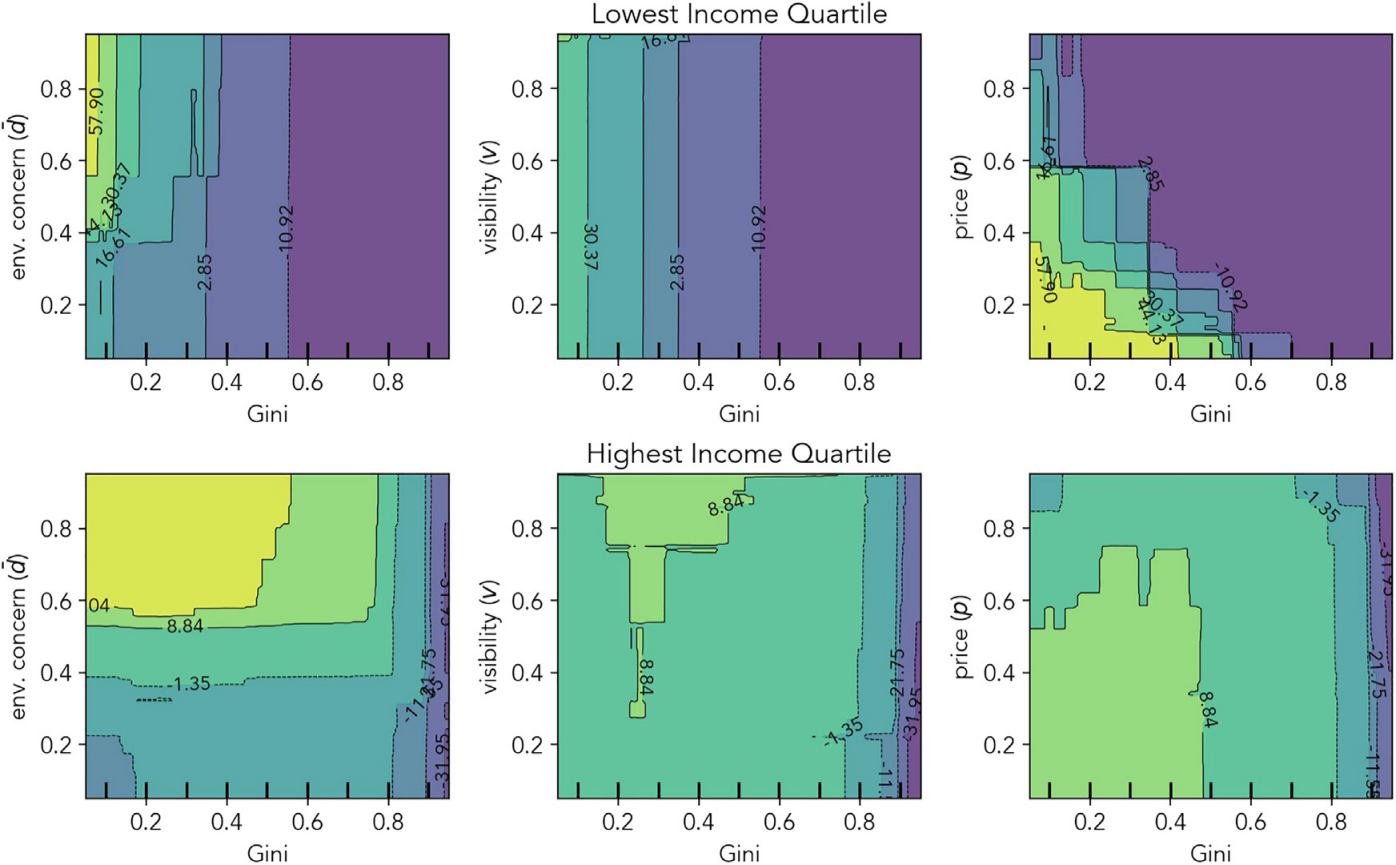
Social desirability more strongly moderates economic deterrents for the highest income group than for the lowest The contour plots show key pairwise interactions on the outcome variable (adoption percentage at t = 10) compared between the bottom and the top income quartile. Plots in the first row are for the bottom quartile and in the second row are for the top quartile. Interactions were estimated using partial dependence functions and thus represent an average effect across all scenarios. Light green indicates a strong positive effect on the adoption ratio at t = 10, dark purple a strong negative effect. Note that the avalanche-like patterns emerge due to the nonlinearity of the effects. As the adoption likelihood across the entire agent population is the outcome, there are “steps” in the partial dependence functions. These indicate how many agents were affected by a change in this parameter. This could be a very low or high percentage, depending on the other parameter settings.

Figure 5 compares key pairwise interactions between the highest and the lowest income quartile. Environmental concern and visibility both interact with inequality, but primarily benefit the top-income quartile. For the highest income quartile, environmental concern dominates inequality, save for high levels of inequality. Visibility makes no difference for the lowest income quartile, but can moderately increase adoption for the highest at low to medium inequality levels. Lastly, product price interacts with inequality by reinforcing its negative effects, particularly for the lowest income quartile.

#### Effects on lower and upper middle-income quartiles

The composition of the middle-income quartiles in terms of behavioral types is highly dependent on the Gini coefficient and, thus, so does the adoption behavior. Besides, price and environmental concern determine the existence and relative frequency of the types in the population as a whole. For the lower and upper middle-income quartiles, we observe that financial factors play a larger role, with inequality being the most important factor regulating adoption and price gaining in significance (relative to the highest income quartile), shown in Figure 4. Social desirability factors, environmental concern and visibility, rank below. The default effect also reduces in importance.

#### Effects on the lowest income quartile

Finally, the lowest income quartile is made up of individuals with behavioral type A (non-adopters) and C (trend followers). Their ratio is determined by price and environmental concern. Trend followers in lower-income groups still adopt when higher-inctome peers adopt, but past a level of inequality or purchase price, this quartile is entirely composed of non-adopters. Therefore, social desirability factors have a smaller (but still positive) effect on the lowest income quartile. Environmental concern, the default effect and product visibility rank below inequality and product price (see Figure 4). Due to the sudden onset of the transition that can be triggered by even low to medium levels of inequality, and the therefore large parameter space characterized by a fixed point at 0% adoption, the degree to which other factors can affect their purchase behavior is limited (see Figure 3). Figure 5 provides further clarification, showing that environmental concern only weakly interacts with inequality to counteract its negative effects in adoption, and visibility not at all for the lowest income quartile. Price, on the other hand, interacts with inequality, enhancing its negative effects.

## Discussion

The findings have significant implications for policies to increase the uptake of environmentally friendly products. Higher and lower-income groups do not respond similarly to changes in social desirability or economic factors. While higher income groups are highly responsive to changes in social desirability, such as environmental concern or how effectively an environmentally friendly product can be used to signal status, lower income groups are held back by financial constraints, especially income and purchase price, and thus not very sensitive to changes in other factors. Therefore, it is necessary for policy design to take into account economic inequality. A policy with a blanket approach that targets everyone in the same way is likely to be less effective, less efficient, and less fair than a tailor-made approach taking into account existing inequalities. Our integrated approach is in line with existing research showing that peer effects can influence adoption behavior,^41^ and that information campaigns aimed at increasing environmental concern can be effective,^14^ while lower income groups are held back by upfront cost.^3^ Furthermore, our results successfully reproduce real-world observations, including the way adoption cascades from higher to lower income groups (see Figure 1) and the regime-dependent effect of income inequality on the adoption of green products (see Figure 2).

For lower-income groups, the highest priority should be removing the obstacle of financial constraints. Income-based subsidies are likely to be effective, where higher subsidies or discounts on eco-friendly products are provided for low-income households. Financing programs with zero or minimal interest for low-income households can help spread the cost of purchasing environmentally friendly products over time. Also, tax exemptions for lower-income groups on the purchase of environmentally friendly products should be considered. Environmentally friendly upgrades can also be paired with utility assistance programs for low-income households, such as programs that install energy-efficient appliances, home insulation, or solar panels at no cost to reduce long-term utility bills. Additionally, policies should ensure that these upgrades are accessible without complex bureaucratic hurdles, making it easier for households to participate. While a simple income-based threshold could be put in place, above which consumers become eligible for the incentive, this might entail issues of fairness and discourage income growth. Therefore, sliding-scale incentive programs should be considered instead. Furthermore, care should be taken that policies do not rely on consumers paying the cost upfront to get it (partially) refunded later, as this has been shown to be ineffective.^43^ Policymakers should also consider integrating education and outreach efforts to raise awareness among lower-income households about available financial assistance and eco-friendly options. Finally, establishing shared, eco-friendly resources in low-income communities would also help reduce individual costs. For example, community solar farms, shared electric vehicles, or cooperative ownership of energy-efficient laundromats. These initiatives could be further supported through partnerships with local businesses and organizations to ensure long-term sustainability and engagement from the community.

Differently, higher-income groups are very receptive to a change in non-monetary switching costs, environmental awareness, or even how visible the product is. Policies that could successfully lower non-monetary switching costs include investing in infrastructure (e.g., building public EV charging infrastructure), streamlined recycling systems for old products and offering standardized installation support when purchasing products like solar panels or home energy systems to reduce complexity. Environmental awareness could be increased with policies such as social media challenges where consumers are encouraged to share their eco-friendly purchases and tag others to join, influencer partnerships, community campaigns (e.g., local workshops) and school outreach programs, where we can encourage adoption by teaching children about sustainability, who then influence parental purchasing decisions, and which shapes the environmental concern of future generations. Product visibility could be increased with neighborhood rewards programs, where local recognition for households adopting eco-friendly upgrades are offered, and providing visibility-enhancing certifications, for example plaques stating “Platinum Energy Efficiency Certified” for homes with extensive eco-upgrades. For high-income consumers, partnering with renowned designers to create limited-edition eco-products, like high-end solar panels or sustainably sourced furniture could be also effective, as well as positioning environmentally friendly products as premium or bespoke.

Our findings also imply that financial assistance programs could be targeted at lower income groups only, especially once the cascade of adoption has started at higher income levels, while more cost-saving policy measures such as nudges can be used for higher income groups to start the cascades. On the other hand, particular care is needed when investing solely in behavioral interventions, as these are most likely to succeed with higher-income groups but not so much with lower-income groups unless their financial constraints are addressed first.

Furthermore, the greater the inequality, the more the adoption behavior of income groups diverges and the more we observe the sorting of different types of adopters by income group. While for higher income groups, price becomes less of a concern, it becomes a significant barrier for lower income groups. Additionally, the existence of high-income individuals means that middle-income individuals exhibit a cascading behavior, where adoption decisions are significantly influenced by peer behavior within their reference groups. This cascade effect is pivotal in determining the community-wide adoption of environmentally friendly products. The fraction of low-income individuals restricts the equilibrium adoption ratio. How these types are distributed across income quartiles critically depends on overall inequality levels and environmental concerns. This also implies that the importance of intervention design increases, as policies need to be more nuanced to be effective at targeting all income groups. Where a country or region with low inequality might be successful with implementing one policy package across the board without carefully designed interventions differentiating by income group, its highly unequal counterpart would see much less success with the same policy package.

Inequality, however, can also positively impact overall adoption levels. In fact, we have shown that in certain scenarios, when product cost is high, it can lead to an increase in adoption. A key question for policy makers and future research is whether increasing overall adoption at the expense of increased adoption inequality is worth it. In the case of certain products, it might help fund R&D and make the product price more accessible in the future, as was the case for the Tesla. However, in certain cases, the product might remain a premium status good.

Furthermore, policy traditionally considers only the tradeoffs of between those who adopt and those who do not, either through cost or some positive intrinsic property of the product (types A and B), where costs and concerns are the only drivers of adoption. New research on peer effects opened the space for contagion of social behavior and, with it, hope that no or small interventions could trigger cascades of adoption, the so-called social tipping.^56^^,^^57^ The interplay between heterogeneity and social tipping is nuanced.^58^ Here, we show that these cascades can be limited by inequality, and the impact of these small interventions highly dampened as it affects adoption of different groups. This finding has significant policy implications. Addressing economic inequality is essential to anticipate the success of small, targeted interventions designed to trigger social tipping points—at least in the context of green product adoption; however, its effects should be examined in other contexts in future research. Where addressing the underlying inequality directly is not feasible due to cost or time constraints, leveling the playing field in the specific context of the relevant policy should be considered. For example, as price is the main barrier for lower income groups in the context of the adoption of green products, policies that remove this barrier could be implemented. Therefore, subsidies, discounts or rebates targeted at lower income groups could reduce adoption inequality and facilitate cascades that reach all income groups. Interventions could even combine financial support with increased product visibility, which may further help overcome barriers created by inequality and trigger the social tipping point. For example, providing targeted incentives for early adopters (e.g., giving away products for free or with large discounts) in disadvantaged groups could generate positive peer effects. These measures ensure that interventions aimed at achieving social tipping points do not falter due to existing economic inequality.

The other implication leading from these findings is that policy evaluation is paramount, and a subgroup analysis should be used to shed light on who a policy intervention ended up targeting. Is the impact of the policy consistent across income groups, or does it differ? If so, who is affected and who is not? These questions must be posed to determine whether the policy’s success is only due to the adoption behavior of higher-income groups and, if so, whether this is a desirable outcome.

The findings of this study also hold significant relevance for advancing sustainable development goals (SDGs), specifically reducing inequalities and climate action (goals 10 and 13) and achieving the 2030 climate goals, specifically achieving equitable access to clean energy solutions. By highlighting the nuanced ways in which income inequality impacts the adoption of environmentally friendly technologies, this research highlights the need for designing equitable and efficient policy interventions and points the way toward achieving those. Tailoring policies to account for economic disparities not only promotes broader adoption and less $CO_{2}$ emissions but also ensures that the benefits of green technologies are equitably distributed across income groups. This alignment with global policy initiatives underscores the importance of addressing inequality as a critical component of the energy transition and should hopefully motivate more research into the complicated relationship between economic inequality and the energy transition.

To conclude, the findings indicate that much more research is needed to understand which policy interventions succeed in encouraging the adoption of environmentally friendly behavior in lower-income groups. Our understanding of this topic is limited, but designing an effective and efficient policy that leads to a just energy transition is crucial.

### Limitations of the study

Our study focuses on the effects of income inequality on adoption behavior of green products across income groups. We consider key factors that play a role in switching to a green(er) alternative, including environmental concern, product visibility, product price, and the default effect. For specific products, additional product characteristics could be taken into account. For example, the amount of CO2 emissions that would be saved by switching, ı.e. a measure of how much greener the new product is, could be included in the environmental concern or be thought of as an intervention. Higher reductions, if known, could lead to greater adoption when environmental concern is high. Another potential product characteristic that could be considered is the long-term economic implications of switching. Households could take into account whether it would help them save money long-term or not, as is the case with solar panels. More generally, some specific type of discounting mechanism could be important. The discounting effect, where future benefits are undervalued, could influence over-exploitation and under-adoption of sustainable practices.^59^^,^^60^ Moreover, the interventions we tested, such as changes in price, concern, and visibility, were applied to the whole population. Targeted interventions can be considered in the future and be optimized for.

A limitation of this study is that it does not explicitly model the long-term effects of product complementarity and substitutability, which are likely to influence the timescales of adoption dynamics. While the individual-level mechanisms, such as financial constraints and social influence, are expected to have consistent effects over time, the interplay between products may alter the persistence or attenuation of these effects. For example, complementary products (e.g., solar panels and energy storage systems) could reinforce long-term adoption trends, while substitutes may shift preferences more rapidly. Future research should incorporate these dynamics to better understand the temporal nature of adoption behavior under different market conditions.

When dealing with longer time scales, allowing for reversibility in product adoption will also be important. Then, considering multiple alternatives and the market of goods where green and non-green suppliers compete for market share and households can choose from a range of substitutes will be important. We would expect that, where there is a larger selection of brown alternatives, switching to a green product might become less likely, as competition drives prices down.

Finally, another interesting avenue for future research would be the heterogeneous pricing of switching costs. For example, a commonly known barrier for switching to green products is oftentimes a lack of infrastructure supporting such goods in lower income areas.^61^ The most known example is EVs, for which the charging stations are usually located in higher-income neighborhoods.^62^ Lower-income groups are therefore either forced to commute to find a charging spot or install one in their own home, not always a possibility. Similarly, for solar panels, renters cannot decide whether to install one on their roof, as they do not own it.^59^ Therefore, it could be insightful to study the effects of heterogeneous switching costs on adoption behavior to understand which policies could be used to mitigate any resulting adoption inequalities.

## Resource availability

### Lead contact

Requests for further information and resources should be directed to and will be fulfilled by the lead contact, Martina Maglicic at martina.maglicic@outlook.com.

### Materials availability

This study did not generate new unique reagents.

### Data and code availability

All original code has been deposited at Zenodo and is publicly available at Zenodo: https://doi.org/10.5281/zenodo.14900781 as of the date of publication. Empirical data have been deposited at Zenodo and are publicly available as of the date of publication at Zenodo: https://doi.org/10.5281/zenodo.14900782. For details, see the key resources table. Any additional information required to reanalyze the data reported in this paper is available from the lead contact upon request.

## Acknowledgments

This work has received funding from the European Union’s 10.13039/501100007601Horizon 2020 research and innovation program under the Marie Skłodowska-Curie grant agreement No 956107, “Economic Policy in Complex Environments (EPOC).” V.V.V. acknowledges funding from ENLENS through the project “The Cost of Large-Scale Transitions: Introducing Effective Targeted Incentives.”

## Author contributions

M.M., conceptualized the study, implemented the simulations, wrote the first version of the paper, discussed the model and its analysis, and edited the paper. V.V.V., provided supervision, performed the analytical analysis of the model, discussed the model and its analysis, and edited the paper.

## Declaration of interests

The authors declare no competing interests.

## STAR★Methods

### Key resources table

**Table:** 

REAGENT or RESOURCE	SOURCE	IDENTIFIER
**Deposited data**		

Gini Index	World Bank^37^(archived on Zenodo)	Zenodo: https://doi.org/10.5281/zenodo.14900782
Tesla’s market share	World Population Review^39^ (archived on Zenodo)	Zenodo: https://doi.org/10.5281/zenodo.14900782
EV market share by country	Canary Media^38^ (archived on Zenodo)	Zenodo: https://doi.org/10.5281/zenodo.14900782
EV registrations in California counties	Atlas EV Hub^27^ (archived on Zenodo)	Zenodo: https://doi.org/10.5281/zenodo.14900782
GDP	US Bureau of Economic Analysis^26^ (archived on Zenodo)	Zenodo: https://doi.org/10.5281/zenodo.14900782

**Software and algorithms**		

Python (Version 3.11.11)	Python Software Foundation^63^	https://www.python.org/
R (Version 4.3.3)	R Foundation^64^	https://www.r-project.org/
Original code	This study	Zenodo: https://doi.org/10.5281/zenodo.14900782

### Method details

#### Model motivation

We drew on the empirical literature on general green purchase behaviour and specific environmentally friendly products such as solar panels, electric vehicles, and heat pumps to design the computational model. In line with the academic literature, we define environmentally friendly products as those with one or more environmental advantages relative to their competitors.^65^^,^^66^^,^^67^ We took into account psychological, social and economic factors. The factors that were selected were based on their measured importance in environmentally friendly product diffusion, i.e., they had to significantly increase, decrease, moderate or mediate product adoption behaviour. Additionally, they had to play a role in creating (in)equality concerning the adoption ratio between income groups. We define the adoption ratio as the percentage of the population that has adopted the product at a specific point in time.

Income itself is one of the key drivers of environmentally friendly product adoption and the resulting inequalities. A number of studies have documented that higher income groups or those living in wealthier regions are also more likely to adopt environmentally friendly products.^23^^,^^68^^,^^69^^,^^70^^,^^71^^,^^72^^,^^73^^,^^74^ This is primarily due to financial constraints and limited purchasing power of lower income groups, but also due to psychological factors, such as mental stress resulting from insufficient financial capacity to cover basic needs,^28^ and physical constraints such as a lower likelihood of owning a home to install solar panels or heat pumps in or lack of access to parking or charging stations.

Peer effects have been shown to significantly boost the adoption ratio of environmentally friendly products and to replicate and reinforce existing inequities.^75^^,^^76^^,^^77^ Peer effects are important to diffusion as they help raise awareness and inform consumers about potential green purchases.^78^ Some have even argued for using peer diffusion strategically to boost the adoption ratio.^79^ However, peer effects have also been shown to replicate existing inequalities.^80^ have shown that individuals are most strongly affected by their neighbours, i.e., individuals from a similar or slightly higher income group. When a social network comprises numerous individuals who lack the financial resources to purchase these products, this creates a vacuum of peer effects and the boost in the adoption ratio that comes with it.^41^ Not only is the peer pressure missing, but so is a wealth of information and awareness of the benefits of environmentally friendly products and any policies that might exist to incentivise their adoption by consumers.

As for purchase motives, two factors are often cited: environmental benefits of the product and its value as a status good, that is, how well a consumer can signal status by owning the environmentally friendly product. Environmental concern, on the one hand, has been consistently reported to significantly influence purchase decisions for green products, including solar panels and EVs.^80^^,^^81^^,^^82^^,^^83^ This is driven, among other things, by social belonging (collectivist rather than individualistic values), self-identification as a green consumer, and perceived social norms.^84^^,^^85^ However, environmental concern is not always the leading motive for green purchase behaviour. With increasing inequality, individuals place more importance on status competition and imitating their wealthier peers, which can drive the demand for electric vehicles and solar panels.^36^^,^^75^^,^^86^ Therefore, product visibility (or conspicuousness) plays a key role when inequality is high. Environmental concern and inequality have also been found to positively interact on inconspicuous environmentally friendly purchases.^87^^,^^88^^,^^89^ Hence, environmental concern can compensate for the lack of product visibility when inequality is high and reinforce the positive impact of inequality on the purchase of conspicuous products. Our model, therefore, incorporates peer effects, environmental concern, and product visibility as motivators of the environmentally friendly purchase decision. Agents must be heterogeneous in income and social networks to study the effects of inequality and social norms.

As agent-based models (ABMs) are an established way of studying patterns that result from interacting and heterogeneous agents, we chose an agent-based modelling approach.^90^ ABMs are computational models based on simple rules that govern the behaviour of decision-making entities or so-called agents.^91^ They have become popular in energy transition modelling, as they allow consumers with heterogeneous characteristics to interact with each other and adapt their behaviour over time.^90^ The bottom-up approach of ABMs also allows patterns to emerge due to agent behaviour, such as self-organization and phase transitions, allowing for a more realistic range of possible system outcomes.^92^ We further derive an analytically tractable case to study the long-term behaviour of many rational individuals under varying economic conditions.

We therefore develop an agent-based model, where agents are consumers who can decide whether or not to purchase an environmentally-friendly product. As discussed in the previous paragraphs, we take existing findings from the literature and make their decision dependent on their environmental concern, income, peer influence and inequality levels in the society. We also model effects of product characteristics noted in the literature, such as product visibility and price. Consumers are heterogeneous in income and the set of peers they are influenced by. As reviewed in the paragraphs above, we assume that product visibility increases in importance with inequality, while the importance of environmental concern decreases. We vary all of these features and observe the adoption levels across income groups and globally. The next section delves into the details of the model.

#### Model specification

The agent-based model consists of three components: agent features, model features and the decision-making function. Agent features describe the characteristics of the decision entities, which are households in this case, and model features define the scenario in which environmentally friendly products diffuse (or not). The decision-making function lays out how these come together when agents decide whether to adopt the environmentally friendly product. The model considers a time frame of 10 years, where each (discrete) time step represents one year. As in reality, households would (re)consider buying an environmentally friendly product in yearly intervals, 1-year time steps were chosen. From a policymaker’s perspective and the climate, the next decade will be crucial in determining whether climate goals will be met, which is why a 10-year time frame was suitable. Further details on the selection procedure of the outcome variable can be found in Section A.

#### Agent features

The decision-makers or agents are households. To isolate the effects of income inequality and peers, we make agents heterogeneous in these aspects but homogeneous in all others. To replicate the current economic inequality, income is unevenly distributed among agents. Driven by status competition, households tend to imitate those in slightly higher income groups.^36^^,^^86^ Therefore, depending on their socioeconomic status, each agent will consider a different group of agents as their peers. We specify this with mathematical notation below.

Let $A=\left\{a_{1},a_{2},\ldots ,a_{n}\right\}$ be the set of all agents (households) in the model, the community. Agents are ordered by ascending income. Each agent *i* has the following characteristics:

• $m_{i}\geq 0$ represents the agent’s yearly income, where 1$m_{i}\leq m_{i+}$.
• $0\leq d_{i}\leq 1$ is the agent’s environmental concern, where 0 is low and 1 is high environmental concern.
• $N_{i}=\left\{a_{i+1},a_{i+2},\ldots ,a_{i+s}\right\}$, where $N_{i}\subseteq A$, is *i*’s reference group, the set of peers that agent *i* compares themselves to, where *s* is the size of this reference group. If i>n−s, agents use the most similar peers as their reference group, $N_{i}=\left\{a_{i+s-n},\ldots ,a_{n}\right\}\setminus \left\{a_{i}\right\}$, or, if i=n, then 1$N_{i}=\left\{a_{i+s-n},\ldots ,a_{n-}\right\}$.
• $o_{it}$ indicates whether agent *i* has purchased the product at time *t*, $o_{it}=1$, or not, $o_{it}=0$.

#### Income distribution among agents

We consider a population with *n* agents. Inequality is exogenous and fixed at a level *δ*, as it is unlikely to substantially change in the timescale of interest (decade). For simplicity, we consider that each community has the same total income of 1, such that $\sum_{i=1}^{n}m_{i}=1$. Thus, we assume a normalized exponential distribution for discrete agents, such that

(Equation 1) $$m_{i}=\frac{e^{\frac{i}{n}\delta }}{Z},\text{where}\;Z=\sum_{j=1}^{n}\;\exp\;\left(\frac{j}{n}\delta \right)\text{is}\;a\;\text{normalization}\;\text{constant.}$$

For large *n*, this distribution has nice properties. Namely, $\min\;m_{i}=\frac{1}{n}\frac{\delta }{e^{\delta }-1}$, $\max\;m_{i}=e^{\delta }\;\min\;m_{i}$, and Gini coefficient^93^^,^^94^^,^^95^ of α=cothδ/2−2/δ. See Methods S1 in the supplemental information for details.

#### Environmentally friendly product features

As for the environmentally friendly product innovation, we distinguish between the following two characteristics:

• $p=\frac{\kappa }{n}$ is the purchase price, computed with cost parameter κ≥0. As the purchase price will be considered relative to income, the factor 1/n avoids the effects coming from the normalization of incomes and varying the purchase price parameter is the same as varying total income levels.
• *v* is the visibility or conspicuousness of the product, i.e., the probability that agent *i* notices that agent *j* in their reference group owns the product. As a probability, 0≤v≤1.

#### Decision-making process

In every time step *t*, each agent *i* is activated in random order, which is reshuffled at each time step, and can choose to purchase the environmentally friendly product. Agents are assumed to be always up to date with the current state of their peers. The decision-making function specifies the process behind this and operates based on a utility with assumptions supported by the psychology and economics literature described in Section A:

• The average level of environmental concern, $\bar{d}$, in the community increases the utility agents gain from purchasing an environmentally friendly product.
• The more unequal the community, the more agents compete for social status.
• The higher the proportion of wealthier individuals that agent *i* observes owning the product, the higher the utility they gain from purchasing the product.
• The higher the income-to-purchase-price ratio for agent *i*, the greater the “pain” that agent *i* feels at purchasing the product. This mimics the probability that the poorer the agent, the more they will have to sacrifice spending elsewhere if they buy the environmentally friendly product.
• Decisions are probabilistic. This is to reflect findings from psychology and neuroscience literature that have shown how decision-making processes are fundamentally stochastic and even with an existing preference, individuals chose their preference with a higher likelihood, but the process is “imprecise”.^96^^,^^97^
• Utility components are defined as a softmax function to allow for non-linearity (for example, peer pressure can grow exponentially with each additional peer that becomes an adopter). Softmax can exhibit increasing, decreasing, or a mix of returns, depending on the shape parameters. The only case where this is not necessary is for the expected disutility. The main text focuses on experiments with identical shape parameters to preserve the interaction between product visibility, environmental concern, and inequality. See Section A for a detailed discussion of the evidence on the direct effects of environmental concern and how it interacts with inequality to affect green consumption. We allow for different shape parameters in the analytical treatments in the supplemental information.

Below, we detail the three components of the decision-making process: environmental concern, product visibility, and purchase price. The average level of environmental concern in the community increases the expected environmental utility $\pi_{E}$ that agents obtain from purchasing the product, through

(Equation 2) $$\pi_{E}\left(d_{i}\right)=\frac{1}{1+e^{\epsilon \left(c_{E}-d_{i}\right)}}\text{,}$$

where $d_{i}=\bar{d}$, and *ϵ* and $c_{E}$ are the relevant response shape parameters (ϵ≥0). This functional form was chosen as environmental concern increases the likelihood of purchasing environmentally friendly products, contributing to the total utility specified in Equation A. Furthermore, as the likelihood of adopting green products correlates strongly with the community’s average level of environmental concern, we assume that agents are homogeneous in their environmental concern for simplification,^89^ meaning this term contributes with a single effective parameter, $\pi_{E}$. Thus, without loss of generality, we fix these shape parameters. Agent *i* also has an expected status utility, $\pi_{S}$, which depends on the visibility of the product, *v*, and the popularity of the product among agents in their reference group, as

(Equation 3) $$\pi_{S}\left(k_{it}\right)=\frac{\alpha }{1+e^{\sigma \left(c_{S}-k_{it}\right)}}\text{,}$$

where σ≥0 and $c_{S}$ are response shape parameters. In the main text, we fix $c_{S}=0.5$, guaranteeing increasing returns until the local majority changes. The value $k_{it}$ is the proportion of wealthier individuals that agent *i* has observed owning the product at time step *t*, i.e.,

(Equation 4) $$k_{it}=v_{p}\frac{\left|O_{it}\right|}{\left|N_{i}\right|}\text{,}$$

where $O_{it}=\left\{j\in N_{i}|o_{jt}=1\right\}$. Status visibility disappears with equality. Notice that visibility always penalizes the fraction of visible adopters of the environmentally friendly technology. Similar to $\pi_{E}$, the functional form reflects the fact that product visibility or its value as a status good encourages individuals to purchase conspicuous environmentally friendly products. Product visibility works in conjunction with peer effects because the more peers own a conspicuous product, the greater the peer pressure to conform. The status utility is substitutable in the total utility (see Equation A). Section A discusses the literature on product visibility and peer effects based on this functional form. The higher the income-to-purchase-price ratio, the more the money spent will “hurt” the agent. We incorporate this into the decision-making process as expected disutility $\pi_{L}$. The expected disutility increases with the ratio

(Equation 5) $$\pi_{L}\left(m_{i},p\right)={\left(\frac{p}{m_{i}}\right)}^{\theta }\text{,}$$

where *θ* is the response shape parameter and θ≥0. The probability of adopting the product at time *t* is derived from the difference in expected utility between owning the product, $\pi_{it}\left(o_{i\left(t+1\right)}=1\right)$, relative to a baseline, $\pi_{it}\left(o_{i\left(t+1\right)}=0\right)$, of not owning it, which can be seen as the all that contributes to the default effect, *f*. $\Delta \pi_{it}=\pi_{it}\left(o_{i\left(t+1\right)}=1\right)-\pi_{it}\left(o_{i\left(t+1\right)}=1\right)$, where

(Equation 6) $$\pi_{it}\left(o_{i\left(t+1\right)}=1\right)=\phi \pi_{E}\left(\bar{d}\right)+\phi \alpha \pi_{S}\left(k_{it}\right)-\gamma \pi_{L}\left(m_{i},c_{p}\right)$$

(Equation 7) $$\pi_{it}\left(o_{i\left(t+1\right)}=1\right)=f$$

In the simulation results, the decision weight associated with status is set to 1, ϕ=1. We additionally make the utility deriving from the environmental concern tradeoff with status by setting, ϕ=1−α. The objective is to leverage more importance from environmental concerns onto status signalling with increasing inequality. As *α* approaches 0 (perfect equality), only environmental concern and financial constraints will influence the purchase decision. On the other hand, as *α* approaches 1 (extreme inequality), status signalling and financial constraints will dominate the decision-making process. For the analytical analysis, we do not impose such a tradeoff. Since *κ* is unbounded, without loss of generality, *γ* is fixed at 1.

Based on the utility function defined in Equation A, the adoption probability is defined as

(Equation 9) $$\text{Prob}\left(o_{i\left(t+1\right)}=1|o_{it}=0\right)=P\left(\Delta \pi_{it}\right)=\frac{1}{1+e^{-\beta \Delta \pi_{i}\left(\bar{d},k_{it},m_{i},p\right)}}\text{,}$$

where *β* controls the variability in the estimates of the utility difference, which a lower *β* increasing the fraction of random decisions. In Annex, we study the case for which decisions are purely rational, β≫1. We consider a relatively short time horizon, which in the context of green technology can be justified as around 10 years and reflects the increasing push of governments towards lowering CO2 emissions. For this, we don’t consider the possibility that agents switch back from owning the environmentally friendly product, so the probability of switching back, $\text{Prob}\left(o_{i\left(t+1\right)}=0|o_{it}=1\right)$ is set to 0. This is also the default in the product diffusion modelling literature.^98^

#### Policy interventions

As set out above, our model considers a range of mechanisms the policymaker could leverage through the implementation of interventions:

• Boosting product visibility through adjustments in product placement or design, plaques that identify owners of the environmentally friendly product, or others;
• Reducing the price of the environmentally friendly product, for example, via subsidies, rebates, investment in R&D, or similar;
• Fostering environmental concern with awareness campaigns or outreach, information and communication programs.

### Quantification and statistical analysis

To evaluate our model systematically and understand why it produces the observed response, we first analyse it within the rational decision limit and then use a framework for ABM analysis^99^ to understand the contribution of the different factors to out-of-equilibrium dynamics.

#### Rational choice analysis

This analysis delves into the analytically tractable case derived from our agent-based model, focusing on the long-term behaviour of many rational individuals under varying economic conditions. The model’s robustness is established through a series of mathematical proofs, detailed in the supplemental information. Please see Theorems S1–S4 for properties of the normalized exponential distribution for discrete agents and Theorems S5–S8 for an analysis of rational choice behaviour. Figure S1 illustrates the terms related to Theorems S5–S8. These proofs and the Figure S1 elucidate the underlying dynamics of income distribution among agents and its implications for adopting environmentally friendly products. In what follows, we focus on the analysis of the out-of-equilibrium results. As mentioned above, for computational tractability and focus on a region of interest, we focus on cases where ϕ=1−α, ϕ=1, and $c_{S}=c_{E}=1/2$.

#### ABM Analysis

We additionally use a framework for ABM analysis,^99^ focusing not on the expected equilibrium adoption but on the dynamics over a given period. This comprehensive approach suggests identifying the model’s features before settling on analysis objectives and, as a last step, deciding on an analysis method. The advantages of this framework are that it provides a systematic approach to selecting model features to analyse and suitable methods to achieve analysis goals. It is also one of the first papers to do so. The next five subsections, therefore, follow the steps recommended by the framework.

#### Outputs of interest

The primary interest of this paper is on the adoption ratio, specifically how fast it increases in the first few years (time steps). For this, we compared four possible outputs of interest: the adoption ratio after 1) 10 years, 2) 20 years and 3) 100 years, and the average growth rate until 100% was reached. Since there were no substantial differences between the four, i.e., the findings remained qualitatively the same, and the 10-year time frame was the most desirable from a policymaker perspective, we chose the adoption ratio after 10 years (1). To account for differences between income groups, we conduct a community-level analysis of all agents and a subgroup analysis. Agents are classified into one of four income quartiles based on their income rank. The lowest 25% are in the first income quartile, those ranked in the $25^{th}-50^{th}$ percentile are in the second income quartile, and so on. We analyse each subgroup individually and then compare results across income quartiles.

#### Goals of the analysis

The primary interest of the study is to study which mechanisms drive inequality in the uptake of environmentally friendly products. Therefore, our analysis focuses on system behaviour due to changing model parameters. This can be done by varying one or more model parameters and estimating the change in system behaviour. In particular, we need to understand the main and interaction effects of the model parameters relevant to our research questions. Because the focus is on inequality and understanding differences between income groups, we conduct this analysis for each income group separately, in addition to a pooled analysis with all agents. We set four goals for the sensitivity analysis^99^:

1) Determine effect sizes and their relative importance (factor prioritization);
2) Determine the direction of the effect i.e. whether features increase or decrease the adoption ratio (direction of change);
3) Quantify the strength and nature of interactions (interaction quantification);
4) Check if the conclusions are robust across analysis approaches and when varying other parameters (robustness check).

#### Features to vary

Since the model is not computationally expensive to run and there aren’t many parameters, we varied all of them on a smaller sample. After that, we screened and fixed parameters that consistently showed no significant effect on the adoption ratio across analysis methods, neither at the community level nor in the subgroup analysis. The paragraph below describes which methods we chose to meet the analysis goals.

#### Methods

We used methods that varied one or more model parameters to estimate the effect size and relative importance of model parameters (analysis goal 1). First, we varied one or two parameters at a time and then visualized the results. This so-called one-factor-at-a-time analysis (OFAT) is particularly useful for detecting non-linear effects and screening for parameters that could be fixed.^100^ In the second step, we systematically varied all parameters to estimate the average effect size across the entire parameter space, also known as Global Sensitivity Analysis.^49^ Due to the bimodal distribution of the data, the commonly used variance-based methods to compute main and interaction effects could not be used here.^101^ Delta and PAWN, which are recommended for estimating the main effects of non-normal output data distributions, were used instead.^55^^,^^102^^,^^103^ As recommended for this approach, we used Latin Hypercube sampling, a quasi-random approach to sampling parameter values in a multidimensional space.^104^ We used machine learning to estimate the direction of the main effects (analysis goal 2) and interaction effects (analysis goal 3). First, we fitted an XGBoost model to the output data (adoption ratio at t=10) with the model parameters as features. We then obtained the direction of the main effects with partial dependence and individual conditional expectation plots. For estimating interaction effects, the first choice would have been to use Sobol’s variance-based method. As mentioned before, this led to biased results due to the bimodal distribution of our output data and thus could not be used. Nor do Delta and PAWN approaches currently support the estimation of interaction effects.^55^^,^^102^^,^^103^ Instead, we used the H-statistic^105^ from the machine learning domain to estimate the strength of interaction effects. The H-statistic measures how much of the prediction’s variation depends on the features’ interaction using partial dependence functions. To analyse and visualise the pairwise interactions that were shown as significant through the H-statistic, we used partial dependence-based feature interaction plots.^106^ To understand the robustness of our results (analysis goal 4), we employ a variety of approaches (Delta, PAWN, machine learning) and check for consistency of results between those.

#### Assignment of values

Table S1 shows the parameter ranges that were sampled. Most parameters were sampled between their minimum and maximum possible value (*p*, $\bar{d}$ and *v*). Where this was not reasonable or informative, we set a wide range. The response shape parameters were varied as to capture the differences between a linear and S-shaped response function. As decreasing parameter values below 5 and increasing past 15 (10 in the case of *θ*) led to limited differences in the response function, we set these as cutoff points. The number of agents was varied to a large enough degree to determine any effects without letting the computation become too expensive. As there was no significant effect from the number of agents on the results, we did not vary *n* any further. The peer group size was set between 10% and 30% of the agent population, as a value below 10% would violate our assumption that peer effects play an important role, and a value beyond 30% would be an unrealistically large comparison group and would not make enough of a distinction between peers and the rest of the population. Purchase price was capped at the value of an average yearly salary, and the rebate was at 50% of the original price, as rebates are often in this range.

## Supplemental information

Document S1. Figure S1, Table S1, Methods S1 and S2, and Theorems S1–S8

Data S1. R and Python scripts for data analysis, related to STAR Methods
