# Supplementary material for: Income inequality in the uptake of environmentally friendly products
Source: iScience. 2025 Mar 25;28(4):112277. doi: 10.1016/j.isci.2025.112277 (PMC12005920; doi:10.1016/j.isci.2025.112277)
Supplement: Document S1. Figure S1, Table S1, Methods S1 and S2, and Theorems S1–S8 [file mmc1.pdf]

iScience, Volume *28*

Supplemental information

Income inequality in the uptake of environmentally friendly products

Martina Maglicic and Vítor V. Vasconcelos

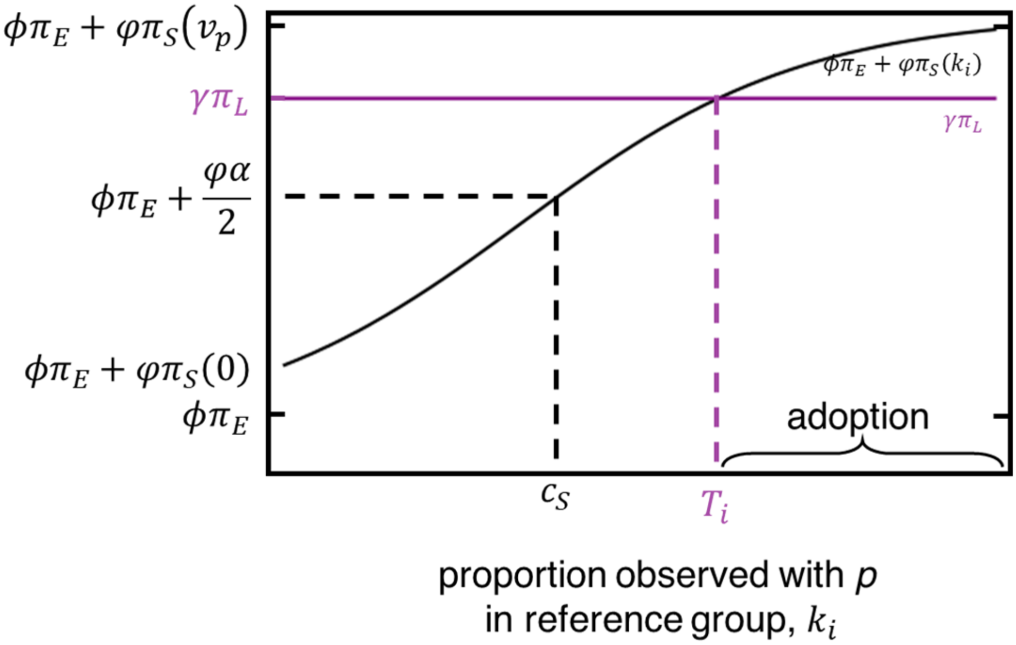

Figure S1: **Terms of payoff as a function of reference group behaviour, related to STAR Methods.** The first two terms in Eq.S3 depend on the agent through their neighbourhood, whereas the last term, representing the evaluation of costs and illustrated in purple, depends on the agent through their wealth. Costs are lower than the environmental and status terms for $k_i > T_i$.

Table S1: **Parameter ranges sampled during OFAT and GSA analysis, related to STAR Methods.** Each parameter is varied one at a time as well as collectively, capturing full effect sizes and interactions.

Parameter	Range	Validation Statement
p (Purchase price / Income levels)	0-1	The higher the price, the lower the probability of adoption. We capture a range of possible product prices, given as a percentage of the average income.
$\bar{d}$ (Env. concern)	0-1	The parameter reflects the positive effect of environmental concern on the adoption of green products. The higher this parameter, the higher the utility of the environmentally friendly product and the higher the probability of adoption. We capture a range of concern levels.
n (Nr. of agents)	200-300	We vary the number of agents in a limited way as this parameter only has a weak effect on our outcome variable.
s (Peer group size)	10-30%	The higher the peer group size, the lower the homophily. We vary the number of peers each consumer is influenced by to capture a variety of peer effects.
v (Product visibility)	0-1	Product visibility has a positive effect on adoption of green products as it allows for peer effects; we vary this in its full range.
Default effect	0-1	The lower the default effect, akin to switching costs, the more likely a consumer is to switch to an alternative product; we vary this in a range comparable to the utility of the product.
δ (Inequality)	0-2	Inequality enhances status utility. We capture the full range of inequality levels, from perfect equality to 1 consumer owning all the wealth.
ϵ (Env. utility response shape parameter)	5-15	The range captures the possibility of linear and non-linear effects of env. concern on adoption probability.
θ (Disutility response shape parameter)	5-10	The range captures the possibility of linear and non-linear effects of relative price on adoption probability.
σ (Status utility response shape parameter)	5-15	The range captures the possibility of linear and non-linear effects of status effects on adoption probability.

Methods S1: Properties of normalized exponential distribution for discrete agents, related to STAR Methods

We consider a population with n agents. We assume a normalized exponential distribution for discrete agents, such that

$$m_i = \frac{e^{\frac{i}{n}\delta}}{Z}, \text{where } Z = \sum_{j=1}^{n} \exp\left(\frac{j}{n}\delta\right) \text{is a normalization constant.} \tag{S1}$$

Theorem S1 (Normalized income)**.** *The distribution of incomes guarantees that for each community* $\sum_{i=1}^{n} m_i = 1$*.*

Proof.

$$\sum_{i=1}^{n} m_i = \sum_{i=1}^{n} \frac{e^{\frac{i}{n}\delta}}{Z} = \frac{1}{Z}\sum_{i=1}^{n} e^{\frac{i}{n}\delta} = \frac{Z}{Z} = 1. \tag{S2}$$

□

Theorem S2 (Minimum wealth)**.** *For large populations,* $\min m_i = \frac{1}{n}\frac{\delta}{e^{\delta}-1}$*.*

Proof. Since $m_i \leq m_{i+1}$, $\min m_i = m_1 = \frac{e^{\frac{\delta}{n}}}{Z}$. Since Z is a geometric sum, it can be computed as $Z = (e^{\delta}-1)\left(1 - \frac{1}{e^{\frac{\delta}{n}}-1}\right)$. We can write it explicitly as a function of δ and n as $\min m_i = \frac{e^{\frac{\delta}{n}}-1}{e^{\delta}-1}$. For large n we can expand in $1/n$ to get $\min m_i = \frac{1}{n}\frac{\delta}{e^{\delta}-1} + O\left(\frac{1}{n^2}\right)$ □

Theorem S3 (Maximum wealth)**.** *For large populations,* $\max m_i = e^{\delta} \min m_i$*.*

Proof. Since $m_i \leq m_{i+1}$, $\max m_i = m_n = \frac{e^{\delta}}{Z}$. Since Z is a geometric sum, it can be computed as $Z = (e^{\delta}-1)\left(1 - \frac{1}{e^{\frac{\delta}{n}}-1}\right)$. We can write it explicitly as a function of δ and n as $\max m_i = e^{\delta}\frac{1-e^{-\frac{\delta}{n}}}{e^{\delta}-1}$. For large n we can expand in $1/n$ to get $\max m_i = \frac{1}{n}\frac{\delta e^{\delta}}{e^{\delta}-1} + O\left(\frac{1}{n^2}\right)$ □

Theorem S4 (Gini coefficient)**.** *For large populations, the Gini coefficient is* $\alpha = \coth\delta/2 - 2/\delta$

Proof. The Gini coefficient is defined in terms of the area between the Lorenz curve and the line of equality. The Lorenz curve for a discrete number of individuals up to individual k is $L(k) = \sum_{l=1}^{k} m_l$, since m_i is normalized to 1. Thus, we can compute $\alpha = \frac{2}{n}\sum_{k=1}^{n}\left(\frac{k}{n} - L(k)\right)$. Replacing leads to $\alpha = \coth\frac{\delta}{n} - \frac{1}{n}\coth\frac{\delta}{2n}$. For large n it becomes $\alpha = \coth\frac{\delta}{2} - \frac{2}{\delta} + O\left(\frac{1}{n^2}\right)$. □

Methods S2: Rational Choice analysis, related to STAR Methods

Individual i considers adopting the product according to the utility of a) an environmental term, $\phi\pi_E$, a status term, $\varphi\pi_S(k_i) = \varphi\alpha/(1+\exp\sigma(c_S - k_i))$, and a cost term, $\gamma\pi_L(m_i) = \gamma(p/m_i)^\theta$, relative to a default option, such that

$$\pi_i = \phi\pi_E + \varphi\pi_S(k_i) - \gamma\pi_L(m_i), \tag{S3}$$

where k_i is the proportion of (visible) neighbours with a higher (non-strict) wealth than i owning an environmentally friendly product, such that $0 \leq k_i \leq v \leq 1$. A rational choice implies that individual i will adopt the product when $\pi_i > 0$ or, equivalently, when $\phi\pi_E + \varphi\pi_S(k_i) > \gamma\pi_L(m_i)$. Figure S1 illustrates these terms. When the two lines cross, there is a critical proportion of wealthier individuals in the reference group of agent i, T_i, above which individual i will adopt the product (and below which they won't).

Theorem S5 (Types of individuals' behaviours)**.** *There are three types of behavioural responses in the population. For an individual i, the following conditions determine the adoption of the new technology:*

- *Type A (Non-Adopters): If* $m_i < p\left(\frac{\phi}{\gamma}\Pi_E + \frac{\varphi\alpha}{\gamma}\frac{1}{1+e^{\sigma(c_S - v)}}\right)^{-\frac{1}{\theta}}$*, then individual i will never adopt (same as* $T_i > 1$*).*
- *Type B (Universal Adopters): If* $m_i \geq p\left(\frac{\phi}{\gamma}\Pi_E + \frac{\varphi\alpha}{\gamma}\frac{1}{1+e^{\sigma c_S}}\right)^{-\frac{1}{\theta}}$*, then individual i will immediately adopt (same as* $T_i \leq 0$*).*
- *Type C (Followers): Otherwise, there is a critical value of* T_i *for* k_i*, above which individual i will adopt and below which individual i will not adopt. Then, individual i may adopt as the neighbourhood changes.*

Proof. Take individual i. They will adopt the new product when $\pi_i > 0$. By replacement of the definitions of π_S and π_L, it results in the condition $\phi\pi_E + \varphi\alpha/(1+\exp\sigma(c_S - k_i)) - \gamma(p/m_i)^\theta > 0$.

The only dynamic variable in this condition is k_i, which takes values in the range $[0, v]$. Since the term $1/(1 + \exp\sigma(c_S - k_i))$ is a monotonically (strictly) increasing function of k_i, its minimum and maximum values are, respectively, $1/(1 + \exp\sigma(c_S)$ and $1/(1 + \exp\sigma(c_S - v)$. The conditions follow immediately.

□

Theorem S6 (Behavior of C types)**.** *All individuals of Type C either all never change or all eventually change*

Proof. If we sort individuals by wealth, we can write $m_i < m_{i+1}$. Since individuals have others of higher wealth as their reference group, Type C individuals have either others of type C or individuals of type B as a reference group (or a combination).

- If individuals of type B exist, i.e., $\max_i m_i \geq p\left(\frac{\phi}{\gamma}\Pi_E + \frac{\varphi\alpha}{\gamma}\frac{1}{1+e^{\sigma c_S}}\right)^{-\frac{1}{\theta}}$, then the wealthiest individual of type C will have a reference group individuals of type B where everyone adopted the product and, thus, by definition of type C, will necessarily adopt the product (otherwise they would be A). From then on, the second highest wealth of type C will now be in the same condition. This will occur recursively until all individuals of type C adopt the product. This condition is equivalent to $e^\delta\frac{\delta}{e^\delta - 1} \geq \kappa\left(\frac{\phi}{\gamma}\Pi_E + \frac{\varphi\alpha[\delta]}{\gamma}\frac{1}{1+e^{\sigma c_S}}\right)^{-\frac{1}{\theta}}$, where $\alpha[\delta] = \coth\frac{\delta}{2} - \frac{2}{\delta}$.

- If individuals of type B do not exist, $\max_i m_i < p\left(\frac{\phi}{\gamma}\Pi_E + \frac{\varphi\alpha}{\gamma}\frac{1}{1+e^{\sigma c_S}}\right)^{-\frac{1}{\theta}}$, then the wealthiest individual of type C will be the wealthiest and we assume an initial condition in which they do not adopt the product. The second wealthiest individual, also of type C, will have a reference group where no one adopted the product and, thus, by definition of type C, will not adopt the product. The third highest will still be in the same condition. This occurs recursively for all individuals of type C, and none will adopt the product.

□

Theorem S7 (Behavior of community)**.** *If individuals of type B exist, B and C types will adopt the technology and As will not. If individuals of type B do not exist, no one will adopt the technology.*

Behavior of community. Follows from previous theorems. □

Theorem S8 (Response to inequality)**.** *Increasing inequality may trigger a cascade. if there Is a tradeoff with environmental concerns, increasing it too much may prevent a cascade. Increasing inequality, under the conditions that the cascade is triggered, reduces total adoption.*

Response to inequality. From theorem 5, we know that individuals with higher wealth adopt first if at all. The triggering of the cascade is controlled by the existence of wealthy enough individuals (Type B), i.e., a high enough $maxm_i$. On the other hand, the extent of the cascade is controlled by the existence of individuals with too little wealth (type A), a low enough $minm_i$. Specifically, the value of $maxm_i$ grows with δ. *In extremis*, when φ is low, the status utility is ignored and δ controls only $maxm_i$, thus, an increase in inequality can induce adoption. On the other hand, increasing inequality, under the conditions that the cascade is triggered, reduces total adoption by increasing the A group, who never adopt. □
